# Extra Health and Health-Related Costs of Living With Frailty Among Rural and Urban Elders in China

**DOI:** 10.1093/geroni/igaa057.255

**Published:** 2020-12-16

**Authors:** Yalu Zhang, Ada Mui

**Affiliations:** 1 Columbia University, Cliffside Park, New York, United States; 2 Columbia University, NEW YORK, New York, United States

## Abstract

Growing attention has been focused on how to improve the affordability and accessibility of healthcare services, especially for elders (aged 55 and above) who have higher levels of medical needs. Following the standard of living approach, which assumes that people’s standard of living would be negatively affected if additional needs (i.e., healthcare) arise at a given level of household income, this secondary research examines elders’ extra health and health-related costs of having chronic diseases and disabilities in rural (n=5,509) and urban (n=3,225) areas of China. Bivariate analyses show there were no significant differences between rural and urban groups in terms of the prevalence of having one or more chronic diseases (56% vs. 58%) and at least one type of disability (15% vs. 13%). Multivariate analyses indicate that living with chronic diseases incurred more extra costs for rural elders than their urban peers, after controlling for individual and household characteristics. On average, rural elders who had at least three chronic medical conditions would spend 108.3% more on medical services than those who had no chronic disease; elders with at least two types of disabilities would spend 59.8% more than those with no disability. The extra health-related costs were boosted when people had at least one type of disability (63.6%), but this was not the case for those who had chronic diseases. Statistical significance was not found among urban elders in China regarding both health and health-related expenditures. The results suggest that rural elders need support to manage their chronic health conditions.

